# The effects of microstructure, Nb content and secondary Ruddlesden–Popper phase on thermoelectric properties in perovskite CaMn_1−*x*_Nb_*x*_O_3_ (*x* = 0–0.10) thin films†

**DOI:** 10.1039/c9ra10007e

**Published:** 2020-02-24

**Authors:** E. Ekström, A. le Febvrier, F. Bourgeois, B. Lundqvist, J. Palisaitis, P. O. Å. Persson, O. Caballero-Calero, M. S. Martín-González, J. Klarbring, S. I. Simak, F. Eriksson, B. Paul, P. Eklund

**Affiliations:** Thin Film Physics Division, Department of Physics, Chemistry and Biology (IFM), Linköping University SE-58183 Linköping Sweden erik.ekstrom@liu.se per.eklund@liu.se; University of Technology of Blois Blois France; Semiconductor Materials Division, Department of Physics, Chemistry and Biology (IFM), Linköping University SE-58183 Linköping Sweden; IMN-Instituto de Micro y Nanotecnología, IMN-CNM, CSIC (CEI UAM+CSIC) Isaac Newton 8, E-28760, Tres Cantos Madrid Spain; Theoretical Physics Division, Department of Physics, Chemistry and Biology (IFM), Linköping University SE-58183 Linköping Sweden

## Abstract

CaMn_1−*x*_Nb_*x*_O_3_ (*x* = 0, 0.5, 0.6, 0.7 and 0.10) thin films have been grown by a two-step sputtering/annealing method. First, rock-salt-structured (Ca,Mn_1−*x*_,Nb_*x*_)O thin films were deposited on 11̄00 sapphire using reactive RF magnetron co-sputtering from elemental targets of Ca, Mn and Nb. The CaMn_1−*x*_Nb_*x*_O_3_ films were then obtained by thermally induced phase transformation from rock-salt-structured (Ca,Mn_1−*x*_Nb_*x*_)O to orthorhombic during post-deposition annealing at 700 °C for 3 h in oxygen flow. The X-ray diffraction patterns of pure CaMnO_3_ showed mixed orientation, while Nb-containing films were epitaxially grown in [101] out of-plane-direction. Scanning transmission electron microscopy showed a Ruddlesden–Popper (R–P) secondary phase in the films, which results in reduction of the electrical and thermal conductivity of CaMn_1−*x*_Nb_*x*_O_3_. The electrical resistivity and Seebeck coefficient of the pure CaMnO_3_ film were measured to 2.7 Ω cm and −270 μV K^−1^ at room temperature, respectively. The electrical resistivity and Seebeck coefficient were reduced by alloying with Nb and was measured to 0.09 Ω cm and −145 μV K^−1^ for *x* = 0.05. Yielding a power factor of 21.5 μW K^−2^ m^−1^ near room temperature, nearly eight times higher than for pure CaMnO_3_ (2.8 μW K^−2^ m^−1^). The power factors for alloyed samples are low compared to other studies on phase-pure material. This is due to high electrical resistivity originating from the secondary R–P phase. The thermal conductivity of the CaMn_1−*x*_Nb_*x*_O_3_ films is low for all samples and is the lowest for *x* = 0.07 and 0.10, determined to 1.6 W m^−1^ K^−1^. The low thermal conductivity is attributed to grain boundary scattering and the secondary R–P phase.

## Abstract 2

Reduction of thermal conductivity of sputtered CaMn_1−*x*_Nb_*x*_O_3_ thin films by secondary Ruddlesden–Popper phase and grain size optimization.

## Graphical abstract

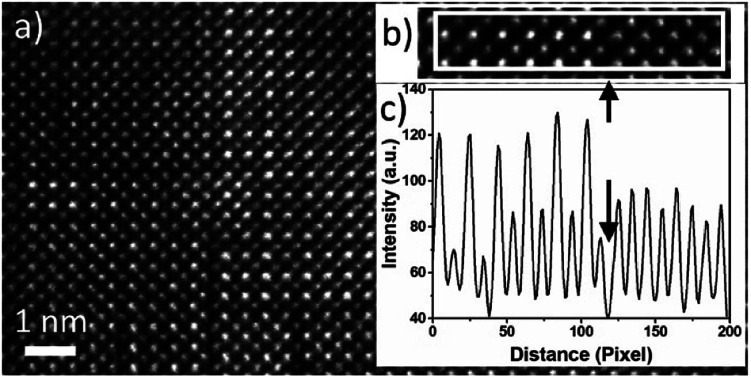

## 1 Introduction

A thermoelectric converter is a solid-state device that can convert heat flux into electrical power and *vice versa*. They have no moving parts and advantages such as high reliability and maintenance-free operation, making them useful as energy sources at remote locations, *e.g.*, in space missions and off-grid power applications.^1,2^ One proposed area of application is waste heat harvesting to generate micro- to milliwatt power for sensors and flexible and wearable devices.^3–5^ Another important application of thin film based thermoelectrics is as Peltier modules for on-chip cooling, where targeted spot cooling can be utilized instead of cooling the whole system.^6–8^ Additionally, thin film thermoelectric modules can also be used as sensors.^9^ Compared to bulk materials, thin films are also of fundamental interest as one can isolate and investigate physical phenomena not readily accessible from bulk polycrystalline materials. For example, the effects of texture, microstructure, and layering, on the thermoelectric properties can be elucidated by thin film studies.

Oxide thermoelectrics are a promising alternative to the conventional thermoelectrics that rely on toxic, expensive, and rare elements and compounds, such as bismuth telluride and lead telluride.^10^ The oxide materials have advantage over conventional thermoelectric materials. They are low-cost, environmentally friendly and can sustain high temperatures.^11^ Despite these advantages, oxide thermoelectrics are not widely used as they often suffer from poor thermoelectric performance either due to high electrical resistivity or, in some cases, high thermal conductivity.^12–16^ The thermoelectric performance of any material system is guided by the dimensionless figure of merit (*ZT*),1
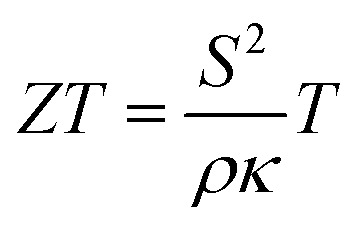
where *S*, *ρ*, *κ* and *T* are Seebeck coefficient, electrical resistivity, thermal conductivity and temperature, respectively.

The power factor, *S*^2^/*ρ*, is more important for applications where the output power from a thermoelectric module is more important than its efficiency, *e.g.* for wearable applications.^5,17^ CaMnO_3_ is reported to exhibit a high Seebeck coefficient, around −200 μV K^−1^ at room temperature.^18^ One strategy to reduce the electrical resistivity and thus enhance the power factor is to alloy CaMnO_3_ with suitable elements to increase the charge carrier concentration. In this regard the partial substitution on Ca site of CaMnO_3_ by lanthanides, and on Mn site by transition metals, *e.g.* Nb and W, have been performed.^19–22^ The substitution of a few percent Mn atoms with Nb atoms is reported to reduce the electrical resistivity, while retaining a high Seebeck coefficient, resulting in the enhancement of the power factor.^18,22^

In the present study, the effect of alloying CaMnO_3_ thin films with Nb, to form CaMn_1−*x*_Nb_*x*_O_3_ (*x* = 0–0.10), is investigated. The thin films are grown by following a two-step sputtering/annealing approach, where radio frequency (RF) reactive magnetron co-sputtering from elemental targets is used together with a post-deposition annealing step.^16,23^ A 5 at% concentration of Nb resulted in a power factor of 21.6 μW K^−2^ m^−1^ at room temperature, which is the highest in this study and an 8 times increase compared to pure CaMnO_3_. This value is low compared to other studies^18,22^ due to the presence of a secondary phase, identified by transmission electron microscopy (TEM) investigations as the Ruddlesden–Popper (R–P) phase.^24^ The R–P phase may act as a scattering centre for both electrons and phonons as we find an increase in the electrical resistivity and a decrease in thermal conductivity.

## 2 Experimental and computational details

CaMn_1−*x*_Nb_*x*_O_3_ films with *x* = 0–0.10 were grown on M-plane (11̄00) Al_2_O_3_ substrates using a two-step approach. The films are labelled Nb0, Nb5, Nb6, Nb7 and Nb10 where the number indicates the value of *x*, *i.e.* the concentration of Nb in the films. (Ca,Mn)O films with increasing Nb content were sputter deposited using reactive RF magnetron co-sputtering, followed by an annealing step to form the CaMnO_3_ phase. The annealing was done at 700 °C under oxygen (O_2_) gas flow for 3 h in a tube furnace. Prior to deposition, the substrates were cleaned in an ultrasonic bath using acetone and ethanol, 10 minutes for each liquid, and blown dry using N_2_. The films were grown in an UHV magnetron sputtering deposition with a background pressure of 4 × 10^−6^ Pa (3 × 10^−8^ Torr). The system is described in more detail elsewhere.^25^ The (Ca,Mn)O + Nb films where grown using elemental Ca (99.5% purity), Mn (99.95% purity) and Nb (99.99% purity) targets. O_2_ and argon (Ar) were used as sputtering gases with an O_2_/(O_2_ + Ar) = 1.5% flow ratio resulting in a 0.29 Pa (2.2 mTorr) deposition pressure. The substrate was kept for 10 min at 400 °C prior to deposition. The deposition time was 30 minutes, resulting in film thicknesses around 250 nm. The magnetrons were operated in RF mode at 50 W for the Ca target and 37 W for the Mn target. The power for the Nb target, run in DC mode, was varied from 0 W for pure (Ca,Mn)O to 3.2 W, 3.7 W, 4.4 W, and 5.2 W for Nb alloyed (Ca,Mn)O. The deposition temperature was 400 °C, and the substrate was kept at a floating potential.

X-ray diffraction (XRD) *θ*–2*θ* scans were performed using a X'Pert PRO powder diffractometer with a copper anode source (Cu Kα, *λ* = 1.54 Å), operated at 45 kV and 40 mA. A 0.5° divergence slit followed by a Bragg-Brentano^HD^ optical module and a 0.5° antiscatter slit on the incident beam path were used. In the diffracted beam path, a 5.0 mm anti scatter slit followed by 0.04 rad Soller slit and a Ni-filter were used. The X'Celerator detector was operated in scanning line mode with a 2.122° active length.

X-ray reflectivity (XRR) measurements were performed using a PANalytical Empyrean diffractometer with a copper anode source (Cu Kα, *λ* = 1.54 Å), operated at 45 kV and 40 mA. A 1/32° divergence slit followed by a hybrid mirror were used on the incident beam path and a triple axis Ge 220 analyser followed by a PIXcel3D detector operated in open detection mode were used in the diffracted beam path.

The structural and chemical constitution of CaMn_1−*x*_Nb_*x*_O_3_ films were explored using high angle annular dark field scanning TEM (HAADF-STEM) imaging, selective area electron diffraction (SAED), and STEM energy-dispersive X-ray spectroscopy (STEM-EDX) characterization approaches in the a double-corrected Linköping FEI Titan^3^ 60-300, operated at 300 kV. The microscope is equipped with a monochromated X-FEG high-brightness gun, high solid angle Super-X EDX detector and an ultrafast Gatan GIF Quantum ERS post-column imaging filter. High-resolution HAADF-(HR)STEM imaging was performed using an optimized 30 mrad convergence angle which provided sub-Ångstrom resolution probes with ∼0.1 nA beam current. STEM-EDX spectrum images of 252 × 252 pixels were acquired for 3 min using ∼0.3 nA beam current.

The morphology was observed with a scanning electron microscope (SEM, LEO Gemini 1550, Zeiss), and the Ca/Mn ratio was determined using energy-dispersive X-ray spectroscopy (EDX) by measuring at several positions on the surface of each sample.

The Seebeck coefficient at room temperature was measured at atmospheric pressure using a homemade Seebeck voltage measurement setup.^26^ The samples were electrically isolated and placed on two copper electrodes separated by 8 mm, which were connected to the surface of the sample (film) and the voltage was measured by a multimeter with a resolution of 0.01 mV. A temperature gradient of 42 °C was applied using a heated metal tip on one electrode, while the other was kept at room temperature. The thermal gradient was calibrated prior to measuring the Seebeck effect by the use of thermocouple on the same spot as the voltage probe is applied. The temperature gradient and the Seebeck voltage were measured after temperature stabilization (holding time: 10 min). The error is estimated to be within 15%. The electrical resistivity of the films were obtained by four-point-probe measurements using a Jandel RM3000 equipment and multiplying the obtained sheet resistance by the film thickness which was obtained by cross sectional SEM imaging and scanning transmission electron microscope (HAADF-STEM) imaging. The error is up to 5% and the main contributor is the thickness estimation.

Thermal conductivity measurements were performed using a time-domain thermoreflectance (TDTR) setup. Gold dots of approximately 200 nm thickness were deposited on the samples using thermal evaporation with an initial 5 nm of nickel for improved adhesion. Gold dot thickness was characterized by a profilometer with an accuracy of ±5 nm. Transient thermoreflectance measurements on the dots were done using a Nd:YAG pulsed laser with a wavelength of 532 nm to heat the sample, and a continuous argon-ion laser with a wavelength of 488 nm to probe the gold on the sample's surface. The pulse duration of the heating laser is 8 ns at full width and half maximum (FWHM) and its low repetition rate of 50 Hz ensures sufficient time for the sample to return to thermal equilibrium between successive pulses. During measurements the sample rests on a temperature controlled chuck, maintaining a stable temperature of 295 K. The collected transients are matched to a heat propagation model and the thermal conductivity of the CaMnO_3_ layer is extracted. The accuracy for the TDTR measurements is typically 10%.

Theoretical calculations on thermal conductivity were performed using density functional theory (DFT) in the framework of the projector augmented-wave (PAW) method as implemented in the Vienna *ab initio* simulation package (VASP).^27–30^ Exchange and correlation effects were treated using the PBEsol functional.^31^ The Kohn–Sham orbitals were expanded in plane waves up to a kinetic energy cut-off of 600 eV and the energy convergence criteria for the electronic self-consistent iterations was set to 10^−6^ eV. We performed Born–Oppenheimer molecular dynamics (BOMD) in the NVT ensemble at *T* = 300 K using a 160 atom supercell constructed as a 2 × 2 × 2 expansion of the orthorhombic unit cell and the *T* = 0 K relaxed structural parameters. The Brillouin zone was sampled at the *Γ* point. The temperature was controlled by a Nosé–Hoover thermostat using the default Nosé mass parameter as set by VASP, and we used a 2 fs time-step. As in Klarbing *et al.*,^32^ the paramagnetic state of CaMnO_3_ (ref. 33) at 300 K was simulated using a disordered local moments (DLM) approach, which here entailed distributing magnetic moments on the Mn ions in the spin up and down channels as a special quasirandom structure (SQS).^34^ The BOMD data was then used to fit a third order effective Hamiltonian using the temperature dependent effective potential (TDEP) method.^35–37^ The phonon thermal conductivity was obtained from an iterative solution to the Boltzmann transport equation, including three-phonon and isotope scattering, on an 11 × 11 × 9 *q*-point grid, where the *z*-direction corresponded to the long direction of the orthorhombic unit cell of CaMnO_3_.^38,39^

## 3 Results and discussion

The as-deposited state ((Ca,Mn)O) of CaMnO_3_ thin films is covered in detail in a previous article.^16^ The addition of Nb changes the texture. XRD and TEM of the as-deposited CaMn_1−*x*_Nb_*x*_O_3_ films are found in the ESI.†

### 3.1 Structural and compositional analysis

The annealed samples were analysed by SEM-EDX and the elemental ratio (Ca : Mn : Nb) obtained in the films are summarised in Table 1. Five set of CaMn_1−*x*_Nb_*x*_O_3_ films with *x* = 0, 0.05, 0.06, 0.07, and 0.10, denoted Nb0, Nb5, Nb6, Nb7, and Nb10, respectively, have been investigated.

**Table 1:** Composition of the CaMn_1−*x*_Nb_*x*_O_3_ films grown with different amounts of Nb. Compositions are normalized so that [Mn] + [Nb] = 1

Sample	EDX
Nb0	Ca_1.01_Mn_1.00_
Nb5	Ca_0.97_Mn_0.95_Nb_0.05_
Nb6	Ca_0.97_Mn_0.94_Nb_0.06_
Nb7	Ca_1.01_Mn_0.93_Nb_0.07_
Nb10	Ca_1.06_Mn_0.90_Nb_0.10_

**Fig. 1:**
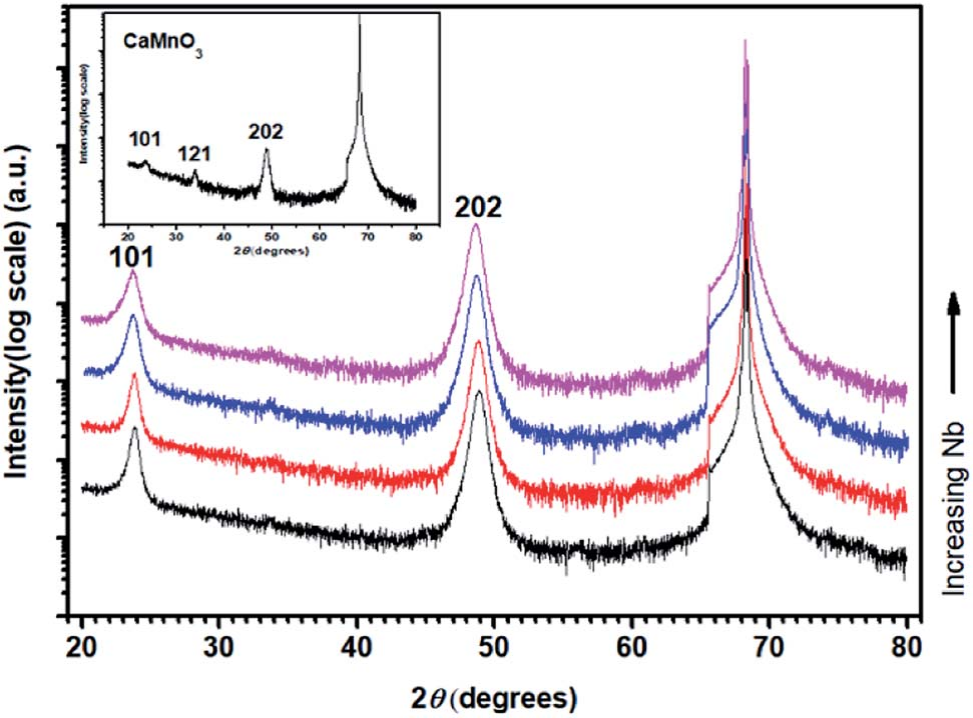
*θ*–2*θ* X-ray diffractogram of post-annealed CaMn_1−*x*_Nb_*x*_O_3_ thin films. Pure CaMnO_3_ in inset.

Fig. 1 shows *θ*–2*θ* X-ray diffractograms of annealed CaMn_1−*x*_Nb_*x*_O_3_ films on M-plane sapphire substrates. The diffraction patterns are matched to the orthorhombic (*Pnma*) structure (PDF 01-076-8574, ICDD, 2010).^40^ The inset diffractogram of pure CaMnO_3_ shows peaks at 23.6°, 33.79° and 48.88°, corresponding to the 101, 121 and 202 peaks of the perovskite CaMnO_3_, respectively. All CaMn_1−*x*_Nb_*x*_O_3_ films have a strong 101 peak at 23.81°, 23.81°, 23.71° and 23.71° and strong 202 peak at 48.86°, 48.81°, 48.69° and 48.61°, with increasing Nb content. The decrease in diffraction angle is a result from substitution of Mn^4+^ ions to Nb^5+^ ions and Mn^3+^ ions where the latter two are larger, causing an expansion of the lattice.

The density of pure (Ca,Mn)O and CaMnO_3_ was determined to 3.90 g cm^−3^ and 4.12 g cm^−3^, respectively, by fitting of XRR measurement data. Both these values are 90% of the theoretical density, 4.35 g cm^−3^ for (Ca,Mn)O and 4.58 g cm^−3^ for CaMnO_3_. The theoretical values are calculated from the XRD diffractograms (PDF 00-037-1497, ICDD, 2010) and (PDF 01-075-6876, ICDD, 2010) for (Ca,Mn)O and (PDF 01-076-8574, ICDD, 2010) for CaMnO_3_. Alloying with the relatively heavy element Nb (in relation to Mn) resulted in an increase in density to 4.40 g cm^−3^, 4.45 g cm^−3^, 4.52 g cm^−3^ and 4.47 g cm^−3^ for Nb5, Nb6, Nb7 and Nb10, respectively.

**Fig. 2:**
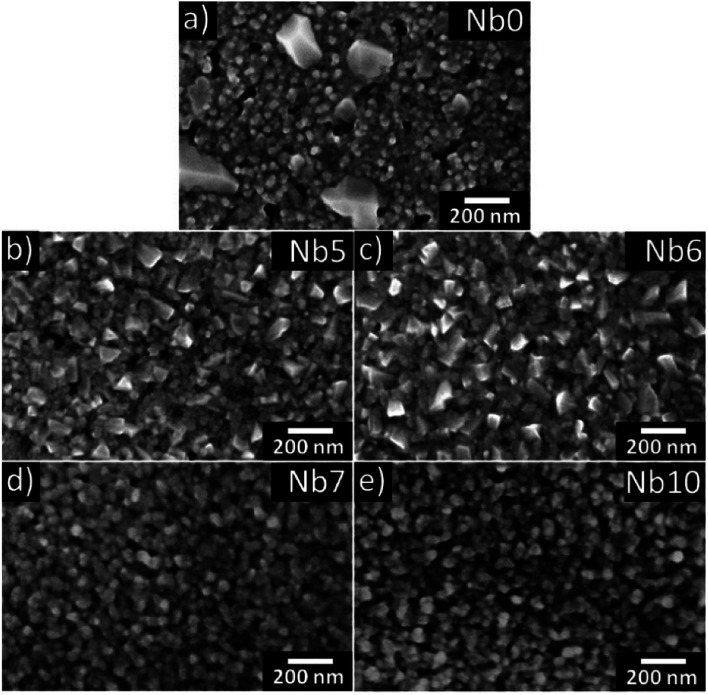
SEM images of annealed CaMn_1−*x*_Nb_*x*_O_3_ films (a–e) are shown where (a), (b), (c), (d) and (e) corresponds to Nb0, Nb5, Nb6, Nb7 and Nb10, respectively.

The SEM micrographs in Fig. 2 show the surface morphology of the annealed CaMnO_3_ (a) and CaMn_1−*x*_Nb_*x*_O_3_ (b–e) films. The pure CaMnO_3_ film has a fine-grained structure with larger facetted inclusions of a secondary orientation corresponding to the 121 orientation observed in XRD (Fig. 1). The addition of Nb in the CaMnO_3_ film reduces the size of these inclusions, as seen in Fig. 2(b) and (c). With higher Nb content the inclusions disappear. It is clear that the films are not single crystalline but have clear domains/grains, where each grain is epitaxially related to the substrate but displaced relative to each other. Apparent porosity is also observed, indicating an underdense film, confirmed by XRR. The apparent grain size in SEM of all samples is similar, Nb5 and Nb6 have slightly larger grains, and typically around 40 nm.

**Fig. 3:**
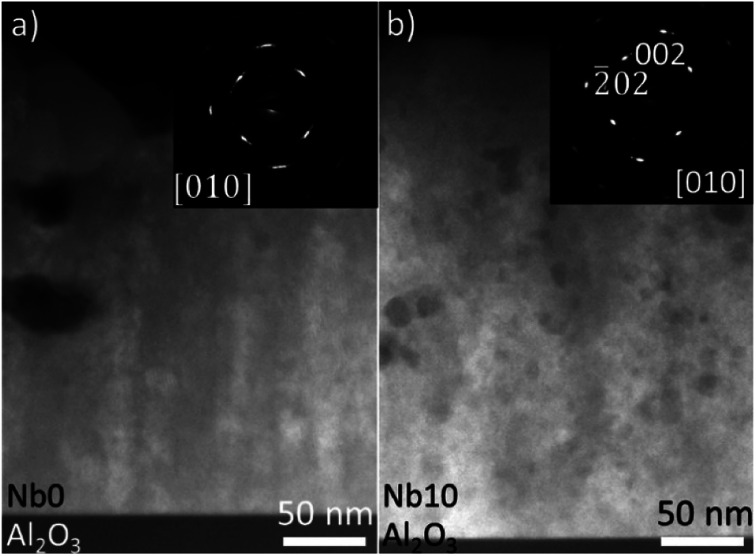
HAADF-STEM images and SAED patterns (inset) of CaMn_1−*x*_Nb_*x*_O_3_ Nb0 and Nb10 for (a) and (b) are shown, respectively.

Fig. 3 shows the HAADF-STEM images acquired from CaMn_1−*x*_Nb_*x*_O_3_ films Nb0 (a) and Nb10 (b). The insets in Fig. 3(a) and (b) are the corresponding SAED patterns. Voids can be seen in (a), and they are present throughout the film. This is in good agreement with the SEM image of pure CaMnO_3_ in Fig. 2(a), where voids are clearly seen on the surface. The SAED pattern shows elongated diffraction spots which indicates mosaicity in the film. The alloyed sample (Nb10) in Fig. 3(b) does not exhibit the same large voids near the surface, however small voids are seen throughout the film. The inset SAED pattern in Fig. 3(b) show more clearly defined spots with a slight broadening in the film pattern originating from mosaicity. The epitaxial relationship is determined from the SAED patterns to CaMn_1−*x*_Nb_*x*_O_3_ (101)‖Al_2_O_3_ (11̄00) and CaMn_1−*x*_Nb_*x*_O_3_ [010]‖Al_2_O_3_ [0001].

**Fig. 4:**
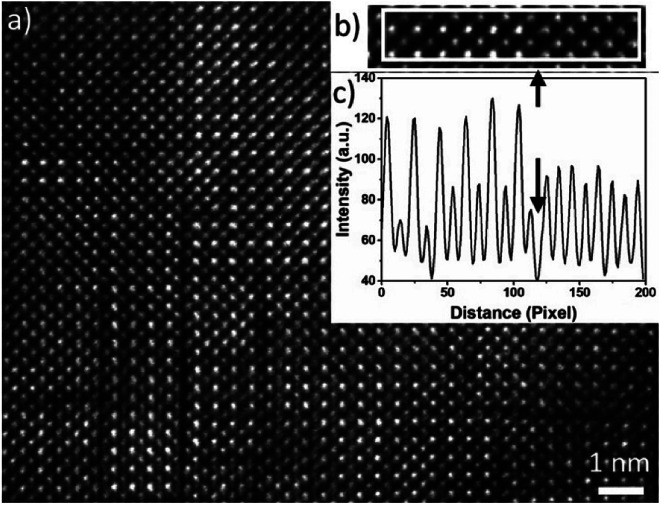
HAADF-HRSTEM image of Nb10 film in (a) and a magnified image is shown in (b) where the white box shows the area where an intensity line scan is taken from. The result of the intensity line scan is shown in (c).

In Fig. 4(a), a HAADF-HRSTEM image acquired from Nb10 film is shown, where dark boundary lines apparently separate nm size domains, both vertically and horizontally. These boundaries are seen in the sample without Nb as well. In addition, two different crystalline phases are observed while analysing the HAADF-STEM intensity variations of different domains. These two phases are separated by a dark line as shown in the line intensity plot in (c) and the corresponding HAADF-STEM image in (b). The left area is the well-known alternating intensity characteristic for a perovskite viewed along the 〈1̄01〉 or 〈010〉 axis (Mn atomic columns showing higher intensity than Ca columns). To the right, the pattern shows more uniform intensity between the atomic columns, clearly seen in the line intensity plot in Fig. 4(c). This feature originates from a R–P (A_*n*+1_B_*n*_O_3*n*+1_) phase.^24,41–43^ The R–P phase is similar to the perovskite and it is built up from ABO_3_ and AO layers. The *n* (*n* = 1, 2, 3…) represent the number of ABO_3_ layers between each AO layer. The stacking sequence results in, when viewed from the [010] direction, mixed Ca and Mn atoms in each atomic column which gives homogeneous intensity variations in the HAADF-STEM image from R–P domains. The crystal lattice is not perfect enough to use the contrast ratio to deduce the value of *n*.

**Fig. 5:**
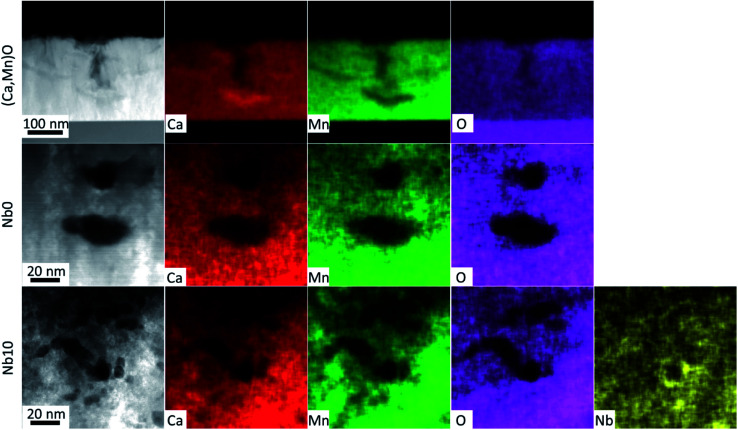
STEM-EDX images of as deposited (Ca,Mn)O, annealed Nb0 film and Nb10 film are shown. The grey scale micrograph corresponds to HAADF-STEM image and red, green, magenta and yellow colour correspond to elemental map of Ca, Mn, O and Nb, respectively.

The EDX maps in Fig. 5 show the distribution of Ca (red), Mn (green), O (magenta) and Nb (yellow) in the films. The EDX maps of the as-deposited pure (Ca,Mn)O film in Fig. 5 show that the observed voids at the surface in HAADF-STEM image in Fig. 3(a) and SEM micrograph in Fig. 2(a) originates from the growth process and not from the annealing step, as they are present in the as-deposited films. The voids are up to 100 nm deep and nucleate by a Ca rich region. These regions are devoid of Mn, but the O signal is constant in the film indicating that the Ca rich region is actually CaO_*x*_. It seems that the incorporation of Nb prevents the agglomeration of Ca as no such regions were observed for Nb-alloyed films and the surface voids disappear with increasing Nb content in the films. The voids in the annealed pure CaMnO_3_ film that are buried inside the film show a uniform distribution of all elements around it. In contrast, the voids in the Nb10 sample show that the Nb concentration is increased in and around the void surfaces, as higher Nb signal is observed in the EDX image in Fig. 5 (yellow).

### 3.2 Thermoelectric properties

**Fig. 6:**
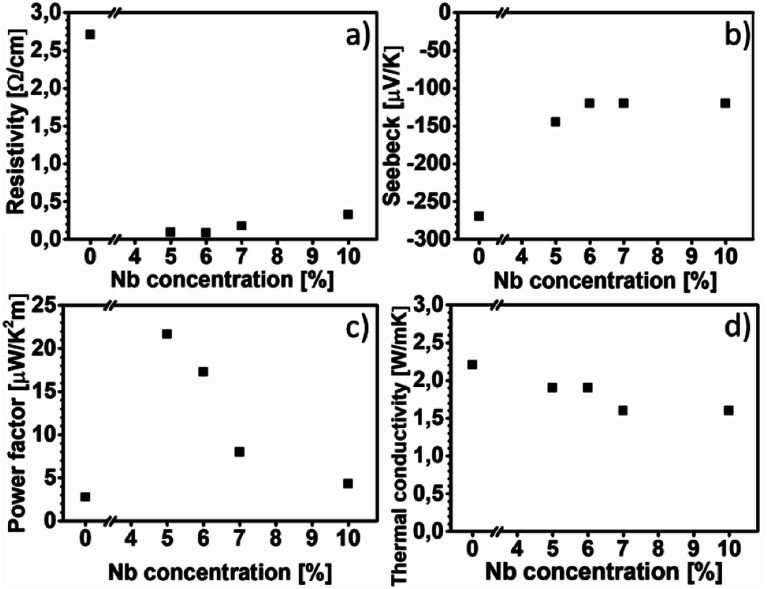
Resistivity, Seebeck coefficient, power factor and thermal conductivity as a function of Nb in the film is shown for (a), (b), (c) and (d), respectively. See the experimental details for information about the accuracy of these measurements.

Fig. 6(a) shows the electrical resistivity values of all the films at room temperature. The electrical resistivity of pure CaMnO_3_ is 2.70 Ω cm, which is comparable to the other reported room temperature values, 1.8 Ω cm (ref. 16) and 8 Ω cm.^18^ The electrical resistivity at room temperature of the Nb-alloyed samples are 0.09 Ω cm, 0.08 Ω cm, 0.18 Ω cm and 0.33 Ω cm with increasing Nb content. These values are comparatively higher than other reports, 2–8 mΩ cm for *x* = 0.04–0.1 (ref. 18) and 10–20 mΩ cm for *x* = 0.05–0.08.^22^ We observe that 5–6% Nb-content results in the lowest electrical resistivity. Further increase in Nb-content beyond 6% leads to an increase in electrical resistivity. The evolution of the electrical resistivity with increasing Nb-content can be tentatively explained as follows. The introduction of pentavalent Nb^5+^ ions at the Mn-site of CaMnO_3_ results in the formation of trivalent Mn^3+^ ions to maintain the charge neutrality. This changes the Mn^4+^–O–Mn^4+^ bonding configuration to Mn^4+^–O–Mn^3+^ bonds, resulting in free electrons in this framework.^44^ It is possible that Mn takes other oxidation states than trivalent as Mn can have a multitude of different oxidation states and we have not confirmed which oxidation state Mn is in. The amount of Mn^4+^–O–Mn^3+^ bonds are related to the amount of Nb that substitute the Mn-site in the CaMnO_3_ films. Thus, an increase in electrical conductivity is expected with the increase in Nb-content in the CaMnO_3_ films, which is observed experimentally in our films at Nb-content up to 6%. At higher Nb content, the effect of introduced scattering centres in the films become dominant over the effect of increased charge carriers, leading to high electrical resistivity.^18,45^ However, it is challenging to quantify the fraction of Nb that is effectively substituting Mn ions, as some of it is concentrated around void surfaces, as observed in EDX. This reduction in effective alloyant amount could affect the electrical resistivity, but most likely not to the extent observed in this work. Instead, the observed R–P phase and boundary lines observed in HAADF-HRSTEM images, seen in Fig. 3 and 4, are more likely the reason for the increased electrical resistivity. There are few studies, both experimental^46–48^ and theoretical,^49,50^ on the electrical properties of Ca_*n*+1_Mn_*n*_O_3*n*+1_, showing that the electrical resistivity increase several orders of magnitude for *n* = 1 compared to the CaMnO_3_ phase. Furthermore, the electrical resistivity is reported to decrease by an order of magnitude with the increase of the value of *n* in the range if *n* = 1 to 4.^46,47,50,51^ Graff and Amouyal reported values for the Ca_*n*+1_Mn_*n*_O_3*n*+1_ phase with different *n* values alloyed with *x* = 4% Nb (Mn_1−*x*_Nb_*x*_).^47^ Our electrical resistivity values for similar alloying level is in the range of *n* = 3 to 5, where the resistivity is 0.097 Ω cm and 0.029 Ω cm, respectively. The reason for the increase in electrical resistivity in the R–P phase lies in the CaO layers which acts as isolating layers, increasing the electrical resistivity.

Fig. 6(b) shows the Seebeck coefficient values of all the films near room temperature. The Seebeck coefficient of pure CaMnO_3_ is measured to −270 μV K^−1^, which is higher than most other reports, typically about −200 μV K^−1^.^18^ The incorporation of Nb in the CaMnO_3_ films reduces their absolute Seebeck coefficient values, resulting in near room temperature value of −145 μV K^−1^ for Nb5 and about −120 μV K^−1^ for the rest of the Nb-alloyed films. Note that the estimated uncertainty of the Seebeck measurement of 15%, *i.e.*, all Nb-containing films are within the same range. The Seebeck values for the alloyed samples are in agreement with other reports, −130 μV K^−1^ to −60 μV K^−1^ for *x* = 0.04 to 0.10 reported by Xu *et al.*^18^ and −130 μV K^−1^ and −120 μV K^−1^ for *x* = 0.05 and *x* = 0.08, respectively, reported by Bocher *et al.*^22^ From the above study it is apparent that the R–P phase act as strong scattering centre for charge carriers, leading to the high electrical resistivity, however with no strong effect on the Seebeck coefficient of the films.

Fig. 6(c) shows the power factor of all the films near room temperature. The pure CaMnO_3_ film exhibits a power factor of 2.7 μW K^−2^ m^−1^. The highest power factor is 21.6 μW K^−2^ m^−1^, obtained from the Nb5 sample. Further increase in Nb content beyond *x* = 0.05 results in a decrease in power factor, as due to the decrease in absolute value of Seebeck coefficient together with an increase of electrical resistivity. Because of high electrical resistivity, the power factor of our alloyed films is about eight times lower than other reported values.^22^

Fig. 6(d) shows thermal conductivity values of all films at room temperature. The pure CaMnO_3_ exhibits the highest thermal conductivity of 2.2 W m^−1^ K^−1^. After alloying with Nb, a reduction in thermal conductivity to 1.9–1.6 W m^−1^ K^−1^ is observed. The electronic contribution to thermal conductivity for all films is negligible, less than 0.01 W m^−1^ K^−1^ at room temperature, calculated using the Wiedemann–Franz law *κ*_e_ = *L*_0_*T*/*ρ* where *L*_0_ is the Lorentz constant. These thermal conductivity values are low for CaMn_1−*x*_Nb_*x*_O_3_, *e.g.*, Kabir *et al.* reported 3.5 W m^−1^ K^−1^ for pure CaMnO_3_ and Xu *et al.* reported 4.15 W m^−1^ K^−1^.^18,21^ Given the presence of R–P phases and boundary lines, possibly containing CaO, a lower thermal conductivity is expected. Graff and Amouyal^47^ reported thermal conductivity values for Ca_*n*+1_MnO_3*n*+1_, both pure and alloyed with Nb. They found that the thermal conductivity, at room temperature, is reduced from 2.7 W m^−1^ K^−1^ for pure CaMnO_3_ to 1.8 W m^−1^ K^−1^ and 0.6 W m^−1^ K^−1^ for *n* = 3 and 2, respectively.

### 3.3 Thermal conductivity calculations

**Fig. 7:**
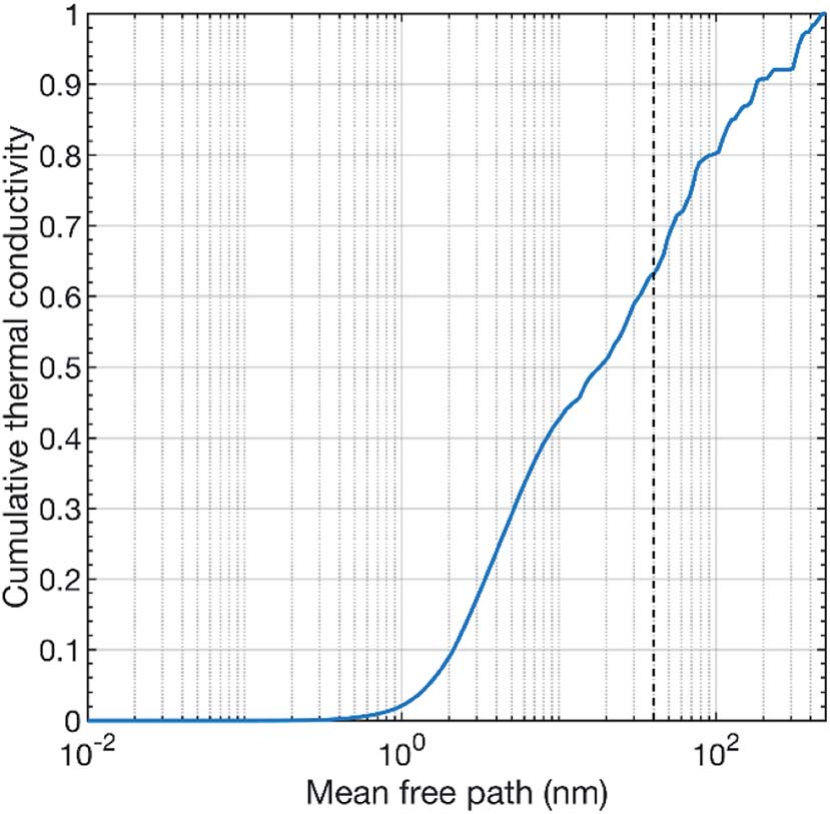
Theoretically calculated cumulative thermal conductivity of pure CaMnO_3_ at 300 K as a function of the phonon mean free path. The vertical dashed line illustrates typical grain size in our samples.

Our theoretical calculations predict a thermal conductivity value of about 7.8 W m^−1^ K^−1^ at 300 K for pure CaMnO_3_. This value is substantially higher than our measured values. However, a theoretical overestimation is expected since our calculations treat a perfect defect free bulk CaMnO_3_.^39^ The films are not free of defects, *e.g.* point defects, line defects and grain boundaries, which can act as strong scattering sources for phonons, reducing thermal conductivity. In our study, the thermal conductivity of the pure CaMnO_3_ film is measured to 2.2 W m^−1^ K^−1^ at room temperature, which is lower than the reported values from other studies.^18,21^ This difference in thermal conductivity can be attributed to the variation in film growth method, resulting in different grain distribution in the film. Conventionally, CaMnO_3_ is grown by solid state reaction method, which results in large grain size, reducing the scattering probability of phonons at grain interfaces. Fig. 7 shows the cumulative thermal conductivity of CaMnO_3_ at 300 K as a function of the mean free path. This shows that the phonons that carry heat have a mean free path between a fraction of a nanometer to roughly 400 nm. The dashed line marks the 40 nm mean free path, which corresponds to the apparent grain size (from SEM, Fig. 2) in our films. The small differences in grain size between samples are neglectable for phonon scattering as changing the grain size by 25% results in less than 5% change in available phonons for efficient scattering, see Fig. 7. More than 35% of the heat-carrying phonons have a mean free path longer than 40 nm. Thus, they should be affected by grain boundary scattering, resulting in a reduction of the thermal conductivity. This means that we see two different contributions to the overall reduction of the thermal conductivity, grain boundary scattering and the R–P phase. However, we also see a decrease in thermal conductivity with increasing Nb content in the films. We propose that the thermal conductivity is reduced by the scattering of phonons on heavy Nb atoms in the lattice where more Nb leads to higher chance of scattering events. Our results thus suggest that the thermal conductivity can be controlled by microstructure optimization or by introducing a R–P secondary phase, where microstructure optimization is the preferred route for thermoelectric applications because the R–P phase reduces the electrical conductivity.

## 4 Conclusions

CaMn_1−*x*_Nb_*x*_O_3_ (*x* = 0, 0.5, 0.6, 0.7 and 0.10) thin films have been synthesised on 11̄00 sapphire substrates by a two-step sputtering/annealing method. We show that it is possible to alloy CaMnO_3_ with Nb by this method. All films are epitaxially grown, as confirmed by STEM analysis. The annealed films exhibit domains, *i.e.*, each grain is epitaxially related to the substrate but displaced relative to each other. A secondary phase was observed and identified to the R–P phase by analysing the intensity variations in the STEM images. This phase is structurally similar to the perovskite but reduces the electrical and thermal conductivity of the films.

The addition of Nb reduces the electrical resistivity of the CaMnO_3_ thin films. The resistivity of the CaMn_1−*x*_Nb_*x*_O_3_ (*x* = 0.06) thin film is the lowest at 0.08 Ω cm near room temperature. Which is nearly 33 times lower than the value for the pure CaMnO_3_ thin film (2.70 Ω cm). The Seebeck coefficient of pure CaMnO_3_ thin film is measured to −270 μV K^−1^ near room temperature, whereas the alloyed films vary within the range −145 μV K^−1^ and −120 μV K^−1^ near room temperature. The highest room temperature power factor value is 21.6 μW K^−2^ m^−1^, obtained from the CaMn_1−*x*_Nb_*x*_O_3_ (*x* = 0.05) film, which is 8 times higher than the pure CaMnO_3_ thin film with a 2.7 μW K^−2^ m^−1^ power factor. Although the electrical resistivity is substantially reduced by Nb alloying, it is comparatively high if compared to other studies on phase-pure material. This is true for the power factor as well (note that the power factor is low compared to other studies).

The thermal conductivity of the pure CaMnO_3_ film is measured to 2.2 W m^−1^ K^−1^ at room temperature, whereas the CaMn_1−*x*_Nb_*x*_O_3_ films decrease the thermal conductivity from 1.9 W m^−1^ K^−1^ to 1.6 W m^−1^ K^−1^ with the increase of Nb content. These thermal conductivity values are lower compared to most other reported values. The low thermal conductivity of our thin film is attributed to the grain boundary scattering of phonons, as due to the low grain size (40 nm) of the films, and a R–P secondary phase in the films.

## Conflicts of interest

There are no conflicts to declare.

## Supplementary Material

RA-010-C9RA10007E-s001
