# Supplementary material for: The effects of microstructure, Nb content and secondary Ruddlesden–Popper phase on thermoelectric properties in perovskite CaMn1−xNbxO3 (x = 0–0.10) thin films
Source: RSC Adv. 2020 Feb 24;10(13):7918–26. doi: 10.1039/c9ra10007e (PMC9049944; doi:10.1039/c9ra10007e)
Supplement: RA-010-C9RA10007E-s001 [file RA-010-C9RA10007E-s001.pdf]

Electronic Supplementary Material (ESI) for RSC Advances.
This journal is © The Royal Society of Chemistry 2020

Supplementary information for

The effects of microstructure, Nb content and secondary Ruddlesden-Popper phase on thermoelectric properties in perovskite $CaMn_{1-x},Nb_xO_3$ (x = 0 – 0.10) thin films

Authors: E. Ekström[a], A. le Febvrier[a], F. Bourgeois[b], B. Lundqvist[c], J. Palisaitis[a], P. O. Å. Persson[a], O. Caballero-Calero[d], M. S. Martín-González[d], J. Klarbring[e], S. I. Simak[e], F. Eriksson[a], B. Paul[a], and P. Eklund[a]

[a] Thin Film Physics Division, Department of Physics, Chemistry and Biology (IFM), Linköping University, SE-58183 Linköping, Sweden

[b] University of Technology of Blois, Blois, France

[c] Semiconductor Materials Division, Department of Physics, Chemistry and Biology (IFM), Linköping University, SE-58183 Linköping, Sweden

[d] IMN-Instituto de Micro y Nanotecnología, IMN-CNM, CSIC (CEI UAM+CSIC) Isaac Newton, 8, E-28760, Tres Cantos, Madrid, Spain

[e] Theoretical Physics Division, Department of Physics, Chemistry and Biology (IFM), Linköping University, SE-58183 Linköping, Sweden

Keywords: PVD, calcium manganate, RF sputtering

S.1. As-deposited Films

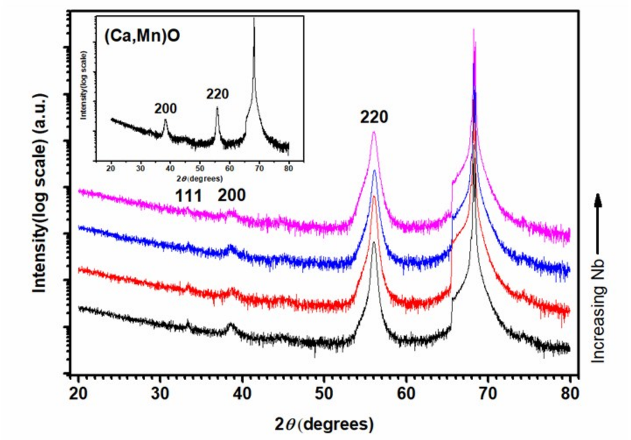

Fig. S1. θ-2θ X-ray diffractogram of as deposited (Ca,Mn)O alloyed with Nb on M-plane sapphire is shown, pure (Ca,Mn)O in the inset.

Figure S1 shows θ-2θ X-ray diffractograms of as-deposited rock-salt structured (Fm$\bar{3}$m) (Ca,Mn)O films alloyed with Nb and grown on M-plane sapphire substrate (substrate peak at 68.2° in 2θ). The inset is a diffractogram of pure (Ca,Mn)O with the same rock salt structure as in (ICDD file 01-077-2373) and shows peaks at 38.39° and 55.88°, corresponding to 200 and 220, respectively. The alloyed films have, with increasing Nb content, a strong 220 peak at 56.04°, 56.05°, 56.09° and 56.01°. A shift to higher angles is expected as NbO has a smaller lattice constant compared to $Ca_{0.5}Mn_{0.5}O$.[1] The 200 and the 111 reflections are also detected, but with low intensity.

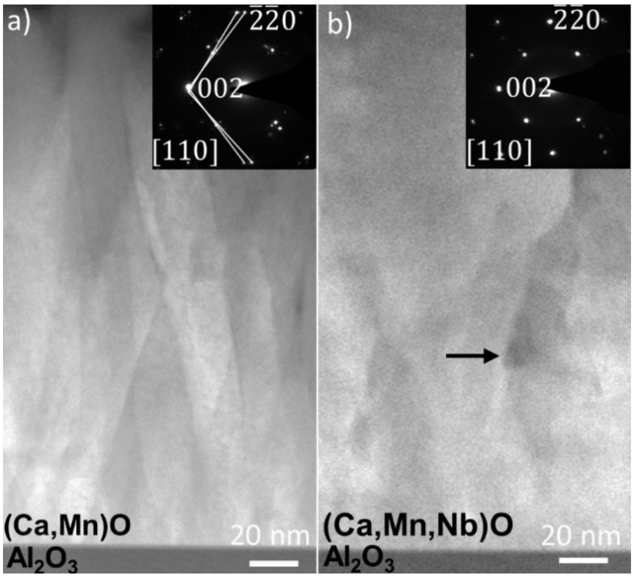

Fig. S2. HAADF-STEM images and SAED patterns (inset) are shown for as-deposited pure and Nb (x = 0.10) alloyed (Ca,Mn)O in (a) and (b), respectively.

Figure S2 show HAADF-STEM images of as-deposited (Ca,Mn)O, both pure (a) and alloyed with 10% Nb (b). Both films show columnar structure where the pure film shows tilted columns with respect to the substrate. These tilts are observable in both the HAADF-STEM image and the SAED pattern, as highlighted by the lines, inset in (a). Empty areas (voids) are observed in the grain boundaries for the Nb alloyed sample (b), indicated by an arrow. Both films are grown with local epitaxy, where the pure film has two competing orientations as observed in the inset X-ray diffractogram in figure S1. However, the SAED pattern does not show any contribution from the observed 100 peak in figure S1. An explanation for this is that the [100] orientation is a minor orientation as the intensity of the 220 peak is almost 5 times stronger compared to the 200, peak and for a fully randomly orientated sample the 200 peak is two times stronger in intensity compared to the 220 peak as seen in ICCD file: 01-077-2373. This means that we expect less contribution from grains grown with an {100}f out of plane orientation and the [100] oriented grains are most likely overgrown by the [110] oriented grains. The crystallographic relationship of the main orientation for both pure and alloyed sample is evaluated to $Ca_{0.5}Mn_{0.5}O$ (110) || Al_2O_3 ($1\bar{1}00$) and $Ca_{0.5}Mn_{0.5}O$ [110] || Al_2O_3 [0001].

S.2. Film density simulations

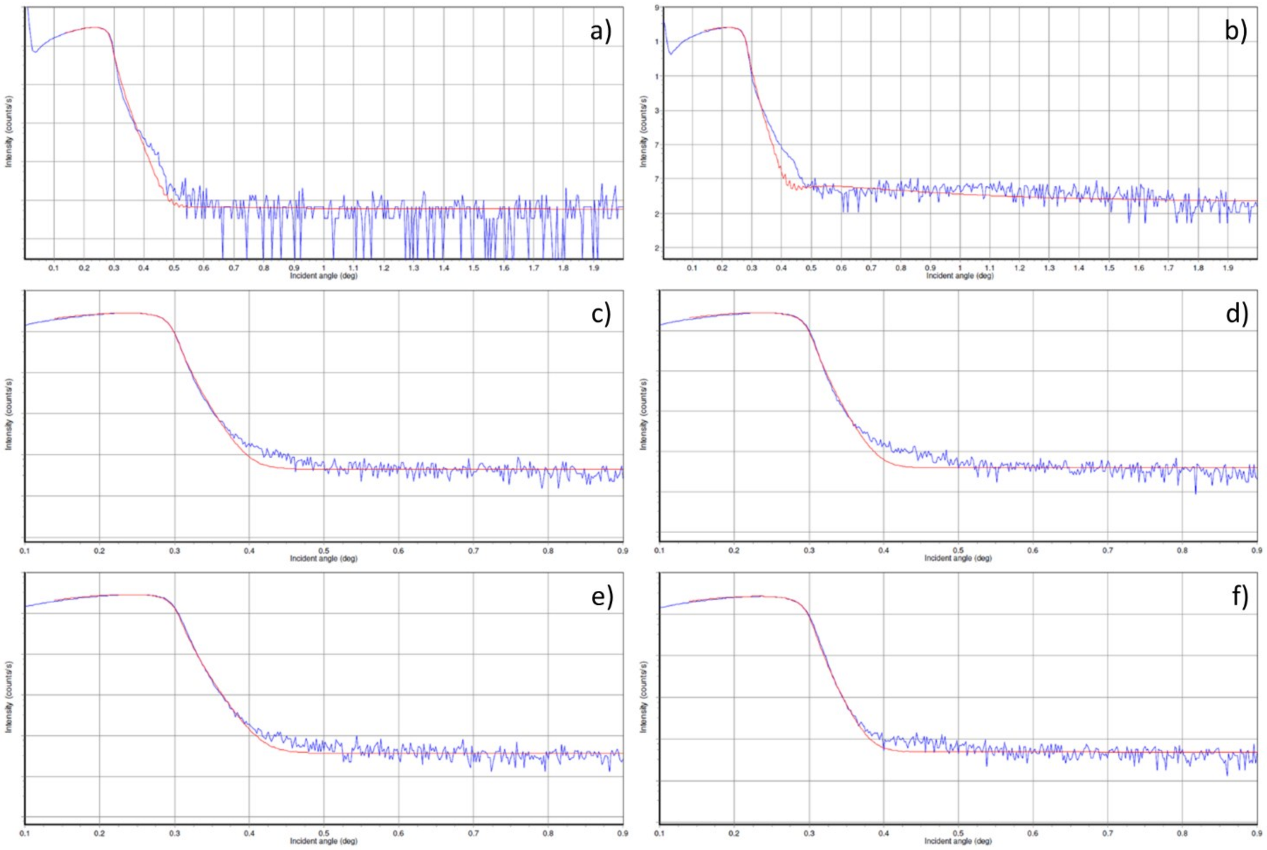

Fig. S3. XRR measurements in blue and fitted simulations in red for as-deposited Nb0 in a) and annealed Nb0, Nb5, Nb6, Nb7 and Nb10 in (b-f), respectively.

The XRR measurements in blue and simulations in red are shown in figure S3 (a-f) for (Ca,Mn)O, Nb0, Nb5, Nb6, Nb7 and Nb10, respectively. The curve simulations are used to determine the density of all films, by utilizing that the critical angle for total external reflection is related to the density. The density for pure films were determined to 4.35 g cm^{-3} for (Ca,Mn)O and 4.58 g cm^{-3} for $CaMnO_3$. The density of the Nb containing were 4.40 g cm^{-3}, 4.45 g cm^{-3}, 4.52 g cm^{-3} and 4.47 g cm^{-3} for Nb5, Nb6, Nb7 and Nb10, respectively.

S.3. Contact problems in Seebeck coefficient measurements

A challenge to achieve a proper contact using the ZEM-3 instrument for Seebeck coefficient and resistivity measurements of the $CaMn_{1-x}Nb_xO_3$ samples was noted during the study. The problem was explored in three different setups. All faced the same problem, where either no contact was achieved or was lost during heating. The resistivity values, when acquired, seemed reliable and comparable to the values acquired from the four point probe Jendel RM3000 instrument as showed in figure S3. The obtained Seebeck coefficient values are somewhat different, because of these contact issues and because the measurement setups differ.

The applied temperature gradients of the three systems compared are different. In one of the home-build measurement system, one side is heated and the other cooled to always keep the median temperature at room temperature. Whereas in the other home built instruments, described in the main article, one side is kept at room temperature and the other heated up, and thus the median temperature at which the Seebeck coefficient is being measured is somehow higher than room temperature.

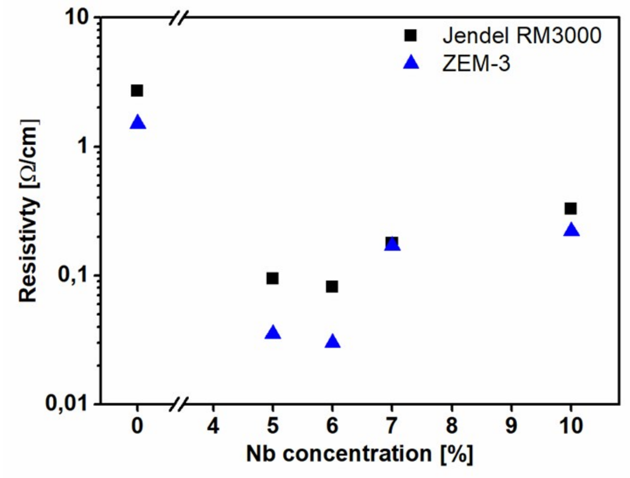

Fig. S4. Comparison of resistivity results from Jendel RM3000 (black squares) and ZEM-3 (blue triangles) instruments.

References

1 A. Jain, S. P. Ong, G. Hautier, W. Chen, W. D. Richards, S. Dacek, S. Cholia, D. Gunter, D. Skinner, G. Ceder and K. A. Persson, *Apl Materials*, 2013, **1**, 011002.
